# Supplementary material for: Resolving the full spectrum of human genome variation using Linked-Reads
Source: Genome Res. 2019 Apr;29(4):635–45. doi: 10.1101/gr.234443.118 (PMC6442396; doi:10.1101/gr.234443.118)
Supplement: Supplemental Material [file supp_gr.234443.118_Supplemental_longranger-2.2.zip › longranger-2.2.tar.bz2!/lr2.2-public/lib/stan/cmdstan/stan/doc/api/html/math_2constants_8hpp_source.html]

Stan: src/stan/math/constants.hpp Source File


|  |  |
| --- | --- |
|  | Stan  2.5.0  probability, sampling & optimization |


- Main Page
- Related Pages
- Namespaces
- Classes
- Files

- File List
- File Members

All Classes Namespaces Files Functions Variables Typedefs Enumerations Enumerator Friends Macros Pages

- src
- stan
- math

constants.hpp

Go to the documentation of this file.

1 #ifndef STAN\_\_MATH\_\_CONSTANTS\_HPP

2 #define STAN\_\_MATH\_\_CONSTANTS\_HPP

3

4 #include <boost/math/constants/constants.hpp>

5

6 namespace stan {

7

8  namespace math {

9

14  const double E = boost::math::constants::e<double>();

15

20  const double SQRT\_2 = std::sqrt(2.0);

21

26  const double INV\_SQRT\_2 = 1.0 / SQRT\_2;

27

32  const double LOG\_2 = std::log(2.0);

33

38  const double LOG\_10 = std::log(10.0);

39

43  const double INFTY = std::numeric\_limits<double>::infinity();

44

48  const double NEGATIVE\_INFTY = - std::numeric\_limits<double>::infinity();

49

53  const double NOT\_A\_NUMBER = std::numeric\_limits<double>::quiet\_NaN();

54

58  const double EPSILON = std::numeric\_limits<double>::epsilon();

59

63  const double NEGATIVE\_EPSILON = - std::numeric\_limits<double>::epsilon();

64

65

66  /\*

67  \* Log pi divided by 4

68  \* \f$ \log \pi / 4 \f$

69  \*/

70  const double LOG\_PI\_OVER\_FOUR = std::log(boost::math::constants::pi<double>()) / 4.0;

71

77  inline double pi() {

78  return boost::math::constants::pi<double>();

79  }

80

86  inline double e() {

87  return E;

88  }

89

95  inline double sqrt2() {

96  return SQRT\_2;

97  }

98

99

105  inline double log10() {

106  return LOG\_10;

107  }

108

114  inline double positive\_infinity() {

115  return INFTY;

116  }

117

123  inline double negative\_infinity() {

124  return NEGATIVE\_INFTY;

125  }

126

132  inline double not\_a\_number() {

133  return NOT\_A\_NUMBER;

134  }

135

142  inline double machine\_precision() {

143  return EPSILON;

144  }

145

146  const double TWO\_OVER\_SQRT\_PI = 2.0 / std::sqrt(boost::math::constants::pi<double>());

147

148  const double NEG\_TWO\_OVER\_SQRT\_PI = -TWO\_OVER\_SQRT\_PI;

149

150  const double INV\_SQRT\_TWO\_PI = 1.0 / std::sqrt(2.0 \* boost::math::constants::pi<double>());

151

152  }

153 }

154

155 #endif

stan::math::LOG\_2

const double LOG\_2

The natural logarithm of 2, .

**Definition:** constants.hpp:32

stan::math::INV\_SQRT\_TWO\_PI

const double INV\_SQRT\_TWO\_PI

**Definition:** constants.hpp:150

stan::math::NOT\_A\_NUMBER

const double NOT\_A\_NUMBER

(Quiet) not-a-number value.

**Definition:** constants.hpp:53

stan::math::LOG\_10

const double LOG\_10

The natural logarithm of 10, .

**Definition:** constants.hpp:38

stan::math::sqrt2

double sqrt2()

Return the square root of two.

**Definition:** constants.hpp:95

stan::math::TWO\_OVER\_SQRT\_PI

const double TWO\_OVER\_SQRT\_PI

**Definition:** constants.hpp:146

stan::math::SQRT\_2

const double SQRT\_2

The value of the square root of 2, .

**Definition:** constants.hpp:20

stan::math::INV\_SQRT\_2

const double INV\_SQRT\_2

The value of 1 over the square root of 2, .

**Definition:** constants.hpp:26

stan::agrad::sqrt

fvar< T > sqrt(const fvar< T > &x)

**Definition:** sqrt.hpp:15

stan::math::EPSILON

const double EPSILON

Smallest positive value.

**Definition:** constants.hpp:58

stan::math::LOG\_PI\_OVER\_FOUR

const double LOG\_PI\_OVER\_FOUR

**Definition:** constants.hpp:70

stan::math::machine\_precision

double machine\_precision()

Returns the difference between 1.0 and the next value representable.

**Definition:** constants.hpp:142

stan::math::NEG\_TWO\_OVER\_SQRT\_PI

const double NEG\_TWO\_OVER\_SQRT\_PI

**Definition:** constants.hpp:148

stan::math::positive\_infinity

double positive\_infinity()

Return positive infinity.

**Definition:** constants.hpp:114

stan::math::e

double e()

Return the base of the natural logarithm.

**Definition:** constants.hpp:86

stan::math::E

const double E

The base of the natural logarithm, .

**Definition:** constants.hpp:14

stan::math::log10

double log10()

Return natural logarithm of ten.

**Definition:** constants.hpp:105

stan::math::INFTY

const double INFTY

Positive infinity.

**Definition:** constants.hpp:43

stan::math::pi

double pi()

Return the value of pi.

**Definition:** constants.hpp:77

stan::math::not\_a\_number

double not\_a\_number()

Return (quiet) not-a-number.

**Definition:** constants.hpp:132

stan::math::NEGATIVE\_INFTY

const double NEGATIVE\_INFTY

Negative infinity.

**Definition:** constants.hpp:48

stan::agrad::log

fvar< T > log(const fvar< T > &x)

**Definition:** log.hpp:15

stan::math::NEGATIVE\_EPSILON

const double NEGATIVE\_EPSILON

Largest negative value (i.e., smallest absolute value).

**Definition:** constants.hpp:63

stan::math::negative\_infinity

double negative\_infinity()

Return negative infinity.

**Definition:** constants.hpp:123


---

[ Stan Home Page ]

*© 2011–2014,
Stan Development Team.*
